# Concurrent Post-Zoster Cutaneous Vasculitis and Acute Calcium Pyrophosphate Deposition Disease (CPPD) in an Elderly Patient: A Case Report

**DOI:** 10.7759/cureus.71614

**Published:** 2024-10-16

**Authors:** Marwa Hallal, Zeina Tannous

**Affiliations:** 1 Department of Dermatology, Faculty of Medicine, Lebanese American University, Byblos, LBN; 2 Department of Dermatology, Lebanese American University Medical Center - Rizk Hospital, Beirut, LBN; 3 Department of Dermatology, Wellman Center for Photomedicine, Massachusetts General Hospital, Harvard Medical School, Boston, USA

**Keywords:** acute calcium pyrophosphate deposition disease, infection, vasculitis, vzv vasculopathy, zoster

## Abstract

Varicella-zoster virus (VZV) can lead to rare complications such as cutaneous vasculitis. We present a unique case of post-zoster cutaneous vasculitis in an 82-year-old male, occurring alongside acute calcium pyrophosphate deposition disease (CPPD), a previously undocumented association. The patient initially presented with a painful zoster rash and hand swelling, treated with oral acyclovir. Persistent swelling led to a diagnosis of CPPD in the wrist, managed with prednisone and colchicine. A biopsy of purplish discoloration at the zoster site confirmed vasculitis, which resolved after treatment. This case underscores the importance of recognizing post-zoster vasculitis, even in immunocompetent individuals, and suggests a potential link with CPPD that warrants further investigation.

## Introduction

Herpes zoster primarily affects older adults, but immunocompromised individuals are at increased risk for atypical and diverse cutaneous manifestations. These may present as ecthymatous, chronic, verrucous, painless, or vasculitic forms, which are rarely seen in the general population [1,2]. Although most cases resolve without major complications, some patients may develop severe sequelae, including postherpetic neuralgia, bacterial superinfection, myelopathy, and meningoencephalitis [3]. While cerebral vasculopathies involving large- and medium-sized vessels have been reported following herpes zoster infection [4], cutaneous vasculitis remains a rare and underreported complication. Cutaneous vasculitis is characterized by inflammation of the blood vessels in the skin, leading to vascular damage and cutaneous manifestations such as palpable purpura, ulcers, and necrosis. The pathophysiology of post-zoster cutaneous vasculitis remains poorly understood but is believed to involve an immune-mediated response to varicella-zoster virus (VZV) reactivation, leading to vascular inflammation and tissue damage [4]. While previous studies have reported rare cases of post-zoster cutaneous vasculitis [1-10], the concurrent occurrence of this condition with acute calcium pyrophosphate deposition disease (CPPD) remains undocumented in the literature. This case report presents a unique clinical scenario, highlighting the potential association between these two conditions. By contributing this case to the limited existing literature, we aim to enhance the understanding of the rare complications associated with varicella-zoster virus infection.

## Case presentation

**Figure 1:**
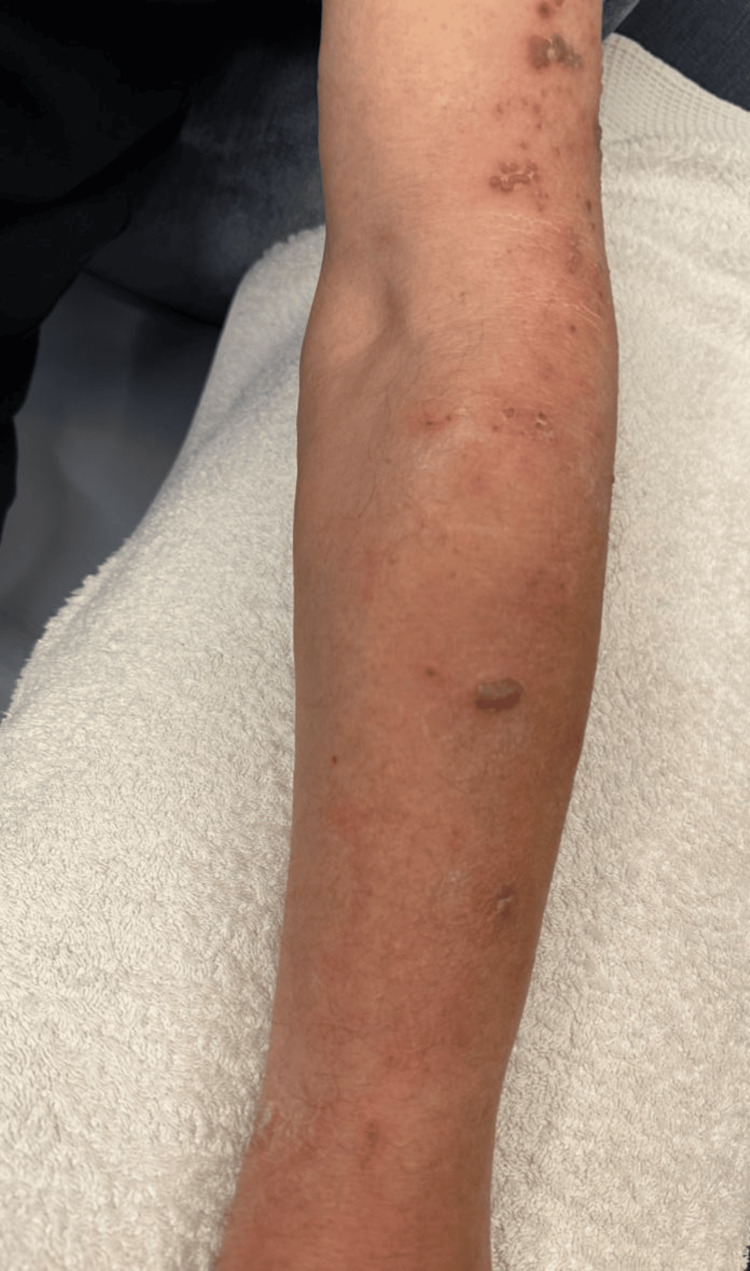
Clinical presentation at initial evaluation showing erythematous papules, vesicles, and bullae localized to the C5 and C6 dermatomes of the left arm.

An 82-year-old male, with a medical history of controlled hypertension, diabetes mellitus, coronary artery disease (status post two stents in 2001 and 2024), and pacemaker implantation, presented to the dermatology clinic. The patient described an onset of burning pain in the posterior neck 2-3 days prior to presentation, which subsequently radiated to the left arm. He reported that the pain was followed by the appearance of red papules and blisters on his left arm, accompanied by swelling in the left hand, which began around the same time. Although the patient has a history of atopic dermatitis, it was well-managed at the time of presentation. He denied any exposure to sick contacts and confirmed a childhood varicella infection but lacked immunization against shingles. He also denied any known immunodeficiency, had a history of occasional alcohol use, and was a non-smoker. Physical examination revealed erythematous papules and vesicles on the patient's left shoulder and forearm (C5/C6 dermatome) (Figure 1) accompanied by erythema, warmth, and swelling in the left hand.

**Figure 2:**
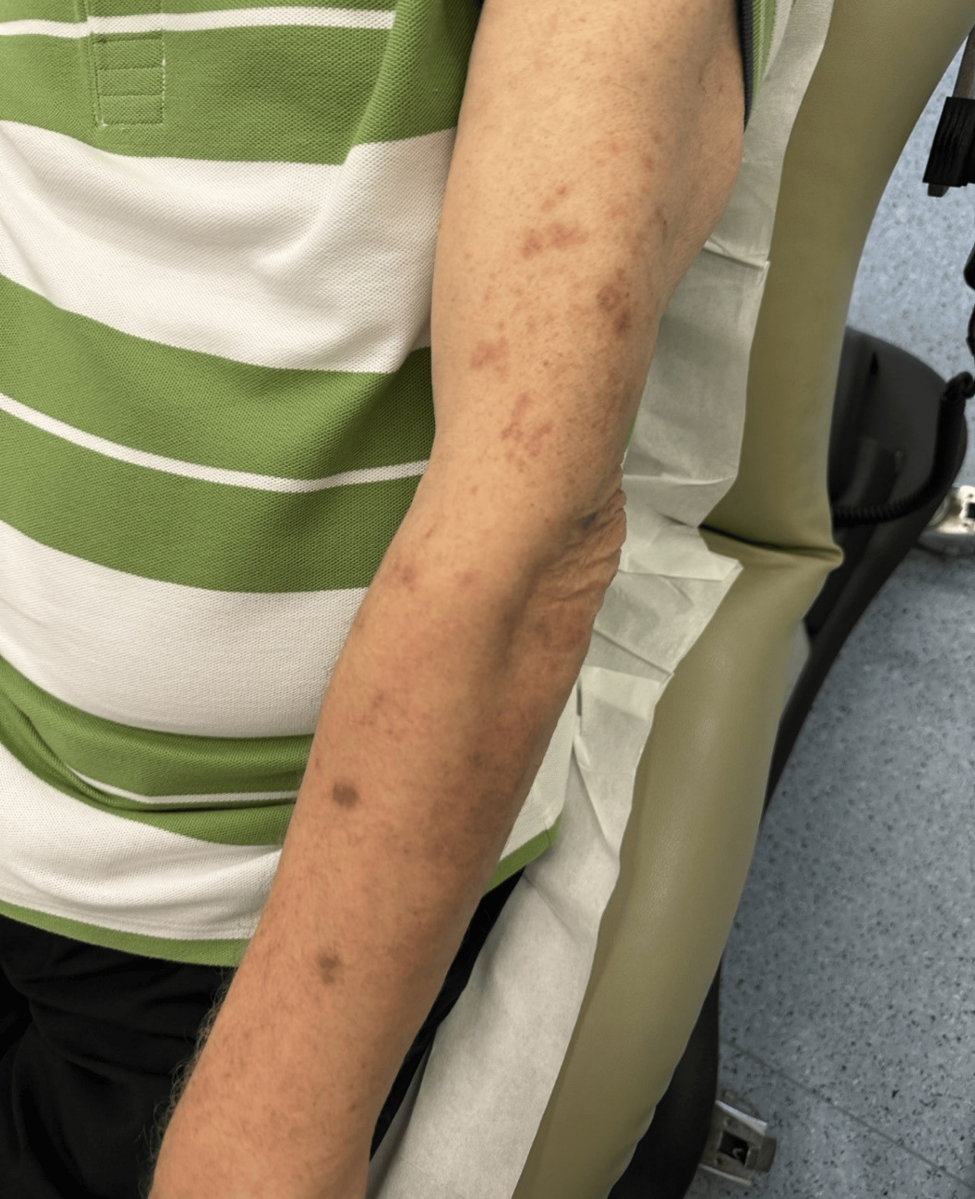
Gradual resolution of the zoster eruption, with residual erythematous macules and post-inflammatory hyperpigmentation.

Despite these symptoms, his vital signs were normal, without fever or hypotension. A clinical diagnosis of herpes zoster (shingles) was established. The patient was initiated on oral acyclovir 800 mg five times daily for a duration of seven days, along with acetaminophen for pain management. Given the swelling, warmth, and redness in the left hand, cellulitis was suspected, and the patient was empirically treated with amoxicillin/clavulanate for seven days. The patient was advised to return to the emergency department if he experienced fever, chills, or clinical deterioration. However, as the hand swelling and erythema did not improve with antibiotics, he was referred to rheumatology. Arthrocentesis and synovial fluid analysis confirmed a diagnosis of calcium pyrophosphate deposition disease (CPPD) in the left wrist. The patient was managed with oral prednisone, initiated at 20 mg per day for two weeks followed by a tapering schedule, alongside colchicine. One week later, he presented to the dermatology clinic with a resolving zoster eruption, characterized by residual erythematous macules and post-inflammatory hyperpigmentation (Figure 2).

**Figure 3:**
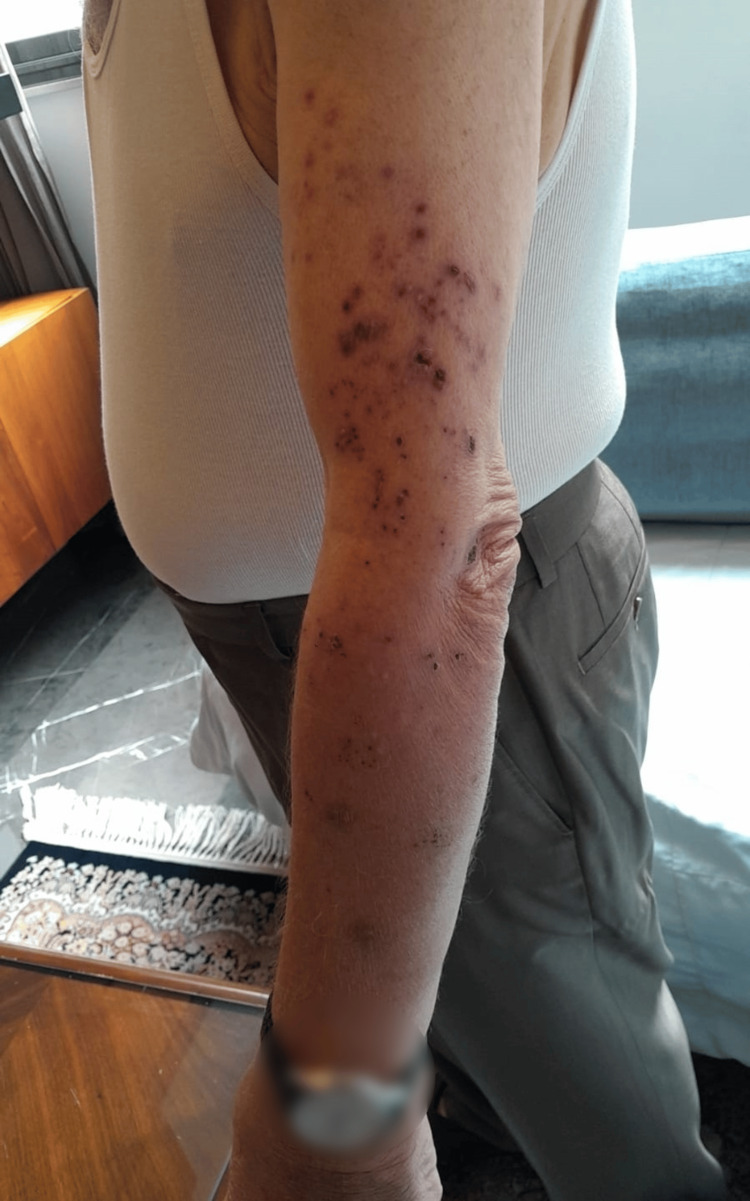
Progression to purpuric macules and patches with crusted erosions in the same area previously affected by zoster.

One month later, the patient exhibited a unique clinical presentation with a purplish transformation of his zoster rash. Physical examination revealed purpuric macules and patches with crusted erosions in the same area previously affected by zoster (Figure 3) alongside improvement in hand swelling following treatment for acute CPPD.

**Figure 4:**
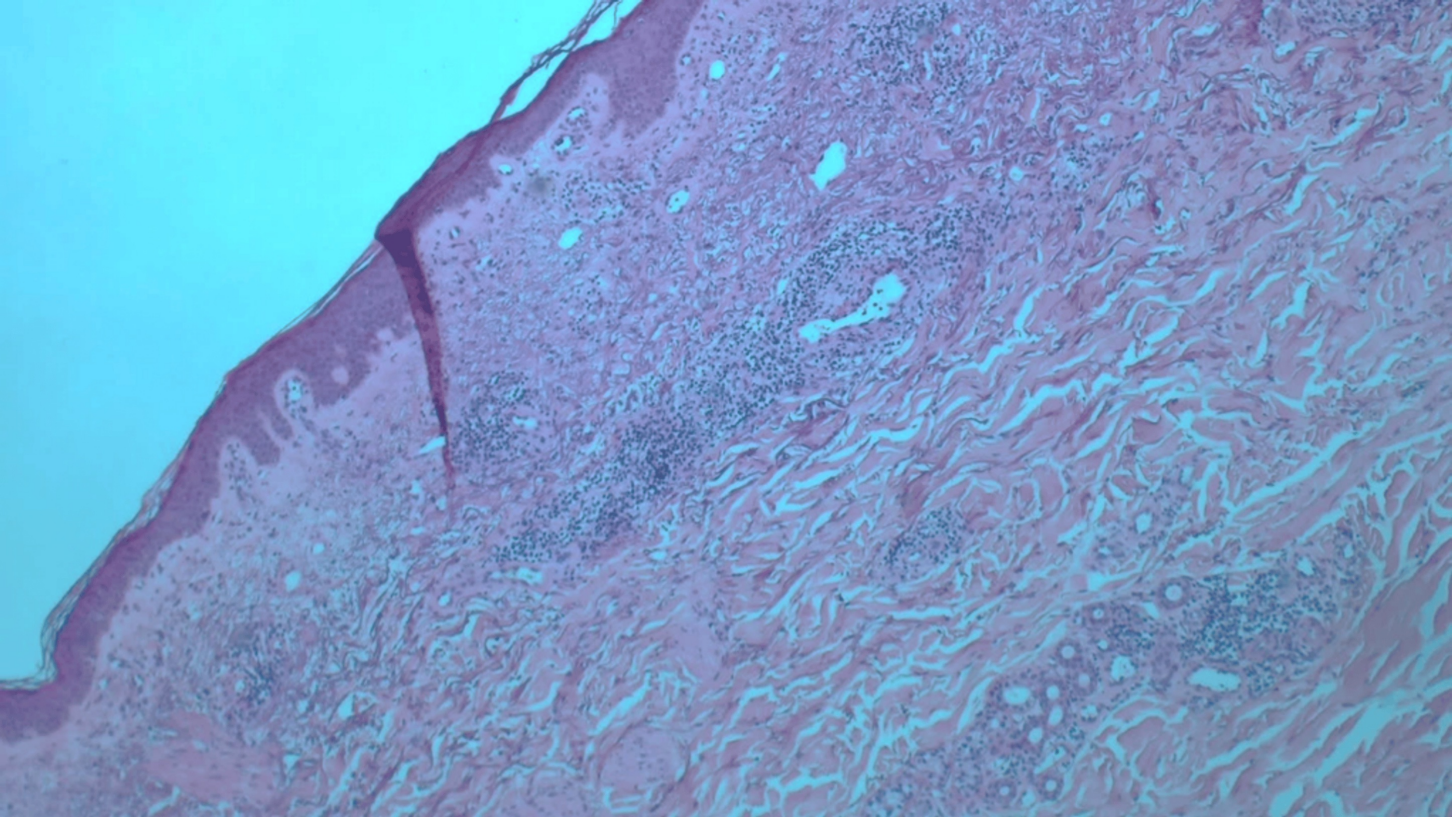
Histopathology of a skin biopsy from the left arm revealing a keratotic epidermis and perivascular inflammation in the upper and mid-dermis, primarily composed of small lymphocytes and some neutrophils with occasional necrotizing vasculitis (hematoxylin and eosin stain).

To rule out a post-zoster lichenoid reaction, a punch biopsy of a purple macule on the left arm was performed. Histopathologic examination with hematoxylin and eosin staining showed keratotic epidermis and perivascular inflammation in the upper and mid-dermis, primarily composed of small lymphocytes and some neutrophils with occasional necrotizing vasculitis (Figure 4).

Based on the clinical presentation of zoster followed by vasculitis on biopsy, a diagnosis of post-zoster cutaneous vasculitis was established. The patient had already received the recommended treatment of oral acyclovir and pain management. Given the asymptomatic nature of the vasculitis and its limited cutaneous involvement, the patient was reassured and monitored closely. The rash subsequently resolved without complications.

## Discussion

Our patient initially presented with classic herpes zoster symptoms, including pain, erythema, vesicles, and bullae, which subsequently crusted over. However, one month later, the patient developed cutaneous vasculitis, a rare complication associated with herpes zoster infection. Although the varicella-zoster virus (VZV) is well-known for its association with a range of vascular complications, particularly those affecting the central nervous system and retina [11], its involvement in the pathogenesis of cutaneous vasculitis remains less clearly defined [2,6]. Post-zoster cutaneous vasculitis can emerge as a complication of herpes zoster, either following the characteristic vesicular rash or, interestingly, preceding the rash with no accompanying pain, as reported by Burgard et al. [3] and Erhard et al. [1]. The study of postherpetic cutaneous vasculitis continues to evolve, particularly concerning its pathophysiology and the connection between herpes zoster reactivation and subsequent vasculitis. Recent literature has underscored the complexity of this condition, with various studies suggesting that leukocytoclastic vasculitis (LCV) can occur as a complication of herpes zoster. For instance, Furuoka et al. (2023) documented a case of segmental cutaneous LCV associated with herpes zoster, which suggests a potential link between viral reactivation and the development of vasculitis [12].

Most reported cases of post-zoster cutaneous vasculitis have been observed in immunocompromised patients, including those receiving treatment for T-cell lymphoma [1] and systemic sarcoidosis [8]. These individuals are particularly susceptible due to the compromised state of their immune systems [1,4,7]. However, our patient was not immunocompromised. This raises the question of whether the prescribed 20 mg prednisone for managing acute CPPD led to transient immunosuppression, thereby facilitating an unusual progression of shingles into cutaneous vasculitis. This possibility is consistent with other case reports linking short-term immunosuppression to such complications [1,4]. In patients with CPPD, short-term immunosuppression significantly increases the risks associated with herpes virus reactivation. Immunosuppressive therapies can impair the host's ability to control latent infections, elevating the likelihood of complications such as postherpetic neuralgia and cutaneous vasculitis following herpes zoster reactivation [13].

Interestingly, our patient's post-zoster cutaneous vasculitis occurred concurrently with acute CPPD in the same extremity, which has not been described previously in the literature. This highlights the potential association between these two conditions. A plausible hypothesis suggests that VZV infection may act as a trigger for the onset or exacerbation of CPPD. Acute herpes zoster is linked to increased levels of pro-inflammatory cytokines, such as IL-1, IL-6, and TNF-α, which are also central to the development of CPPD [14,15]. The release of these cytokines during viral infection could promote localized inflammation and immune activation in surrounding tissues, potentially aggravating preexisting CPPD or triggering a new episode in predisposed individuals. Early recognition of post-zoster cutaneous vasculitis is crucial to prevent severe complications, including ulceration, necrosis, and secondary infections [8,10]. The condition can be easily misdiagnosed due to its similarity to other skin disorders such as leukocytoclastic vasculitis and pyoderma gangrenosum [9], emphasizing the need for prompt and accurate diagnosis.

## Conclusions

In conclusion, postherpetic cutaneous vasculitis presents a complex interplay of viral infections, immune responses, and therapeutic interventions. Recent advancements in understanding its pathophysiology and treatment, alongside the implications of calcium pyrophosphate deposition disease (CPPD) and immunosuppression, highlight the importance of ongoing research and clinical vigilance in effectively managing this condition. Further studies are essential to enhance our understanding and improve patient outcomes.
